# Unsupervised Learning Facilitates Neural Coordination Across the Functional Clusters of the *C. elegans* Connectome

**DOI:** 10.3389/frobt.2020.00040

**Published:** 2020-04-02

**Authors:** Alejandro Morales, Tom Froese

**Affiliations:** ^1^Embodied Cognitive Science Unit, Okinawa Institute of Science and Technology Graduate University, Okinawa, Japan; ^2^Computer Science and Engineering Postgraduate Program, National Autonomous University of Mexico, Mexico City, Mexico

**Keywords:** artificial neural networks, self-organization, Hebbian learning, self-modeling, complex adaptive systems, Hopfield networks, artificial life, computational neuroscience

## Abstract

Modeling of complex adaptive systems has revealed a still poorly understood benefit of unsupervised learning: when neural networks are enabled to form an associative memory of a large set of their own attractor configurations, they begin to reorganize their connectivity in a direction that minimizes the coordination constraints posed by the initial network architecture. This self-optimization process has been replicated in various neural network formalisms, but it is still unclear whether it can be applied to biologically more realistic network topologies and scaled up to larger networks. Here we continue our efforts to respond to these challenges by demonstrating the process on the connectome of the widely studied nematode worm *C. elegans*. We extend our previous work by considering the contributions made by hierarchical partitions of the connectome that form functional clusters, and we explore possible beneficial effects of inter-cluster inhibitory connections. We conclude that the self-optimization process can be applied to neural network topologies characterized by greater biological realism, and that long-range inhibitory connections can facilitate the generalization capacity of the process.

## 1. Introduction

The brain consists of a vast number of interacting elements. An important research question is how this complex adaptive system manages to give rise to large-scale coordination in the service of cognition, especially in the absence of a central controller or explicit knowledge of what would be the best neural connectivity. A promising approach is therefore the study of self-organization in artificial neural networks. Watson et al. (2011b) developed a self-optimization algorithm in Hopfield neural networks able to form associative memory of its attractor configurations through unsupervised learning of the Hebbian variety. This causes the networks to begin to reorganize their connectivity in a direction that minimizes the neural coordination constraints posed by the initial network architecture.

Previous work with this algorithm has been done using fully-connected networks, but without self-connections, and only with non-directed connections constrained to symmetric weights that are assigned in a random or highly modular manner (Watson et al., 2011a,c). More recently, self-optimization has also been demonstrated in the case of continuous activation functions (Zarco and Froese, 2018a,b). This shows that the self-optimization process might be more generally applicable. Nevertheless, a concern with this work is that these network topologies are too artificial compared with those of actual neural networks. Accordingly, we propose that it would be more meaningful to employ the connectome of a real organism in order to better assess the scope of self-optimization.

A particularly suitable connectome comes from the nematode worm, *Caenorhabditis elegans*. This worm is one-millimeter-long and consists of only 959 cells, of which 302 belong to the nervous system. *C. elegans* is relevant in this research because it is a reference model in biology (White et al., 1986; Walker et al., 2000; Girard et al., 2006). It was the first multicellular organism whose genome has been sequenced in its entirety, as well as the first animal whose neural connections, called connectome, has been completed. *C. elegans* has also been studied in the field of artificial life using agent-based modeling (Izquierdo and Beer, 2015; Izquierdo, 2018).

In recent work, we demonstrated self-optimization in the *C. elegans* connectome (Morales and Froese, 2019), by turning it into a Hopfield neural network that captures the connectome's directed multigraph topology including its self-connections. We set two simulation experiments: (1) we ran the self-optimization algorithm with only excitatory (positive) connections, and (2) with 30% inhibitory (negative) connections arbitrarily assigned in a homogeneous fashion at the beginning of the algorithm. Under these conditions the *C. elegans* connectome showed a tendency to optimize its own connectivity, but more so in case (1). The addition of inhibitory synapses increased the difficulty of learning to find attractors with optimal neural coordination, and there remained a broader spread of attractors even after convergence. We hypothesize that this has to do with how coordination happens in functionally related neurons within clusters of the connectome.

Here we explore the possibility that this poor performance can be overcome by making inhibitory connections more concentrated between clusters, thereby also making our analysis more biologically plausible. We ran the self-optimization procedure in the whole *C. elegans* connectome, but also separately for each of the hierarchically organized functional clusters. We performed two sets of simulation experiments: (1) we arbitrarily assigned 30% inhibitory connections to local connections within each cluster, and ran self-optimization on each of the clusters as an independent network, and (2) we applied 30% of inhibitory connections to the whole connectome but restricted them to long-range inter-cluster connections, and ran the process on the entire connectome while also monitoring neural coordination within clusters.

The key finding of these simulation experiments is that the poor performance associated with the introduction of inhibitory connections can be successfully overcome by focusing inhibition to connections between clusters. This is the case both in terms of the number of attractors found and their energy levels: the process tends to converge on a more refined set of more optimal attractors, including attractors that normally would not be found by the network prior to self-optimization. Interestingly, while this capacity to generalize to better attractors is also noticeable in the clusters when self-optimization is run on them independently, generalization is less marked when they are evaluated while embedded into the whole network—even though in the latter case they tend to converge on lower energy values because they do not have to overcome the added coordination constraints introduced by local inhibitory connections. This suggests that generalization to better attractor configurations is a property of the whole network, rather than being a simple reflection of generalization occurring at the level of local clusters.

## 2. Methods

### 2.1. The Connectome

We ran the self-optimization algorithm in the connectome published by Jarrell et al. (2012). The database contains hermaphrodite neural system information (because males arise infrequently, at 0.1%), such as connection direction, type of connection (synapse or gap junction), and the number of connections between neurons. We translate the connectome into a directed multigraph, with neurons as nodes and connections as edges. Chemical synapses are modeled as single-directed links between neurons (for example, *A* → *B* indicates that neuron *A* is presynaptic to neuron *B*, and *B* is postsynaptic to *A*). Gap junctions are represented in the model as double-linked neurons (if two neurons, *C* and *D*, have a gap junction between them, there are two links: *C* → *D* and *D* → *C*).

We assigned binary activation states (−1, 1) to neurons. The number of connections between neurons was assigned as the weight of each edge, normalized in the interval (0, 1). Both links in gap junctions were assigned the same weight, and values vary between 1 and 81 before normalization (and form a power law). Therefore, we clip to 1 the 15 high weight values, which we determine with an arbitrary cut-off of weights greater than 44. Reduction of this outliers broadens the state-space explorations during the self-optimization.

We did not also consider pharyngeal neurons because they belong to another independent neural system (Albertson and Thompson, 1976). Only 279 neurons are taken into account, with 5,588 connections. This differs from the number in our previous paper (282 neurons and 5,611 connections) because here we follow Sohn et al. (2011) in removing the neurons *VC*6, *CANR*, and *CANL* which do not have obvious connections.

Sohn et al. (2011) proposed a modular organization of the *C. elegans* connectome in five clusters based on a constraint community detection method for directed, weighted networks. This model shows hierarchical relationships between the clusters that define systemic cooperation between circuits with identified biological functions (mechanosensation, chemosensation, and navigation). This division also considers bilateral neural pairs present in the connectome so that the members of a pair should not be assigned to different structural clusters. There are two big clusters named 1 and 2. Smaller cluster names have hierarchical branch names: 1 (or 2) represents a big cluster branch in the left digit and small cluster branching is called 1 (or two rightward) in the right digit. Table 1 shows the basic information of each cluster. The authors also observed many ties between the clusters depended on hierarchical proximity. Cluster 11, 12, and 13 comprise a big cluster, and cluster 21 and 22 formed another grand cluster.

**Table 1:** This table contains cluster information from the partition of Sohn et al. (2011), including the number of nodes and edges, average degree, and average shortest path of each cluster.

**Cluster name**	**No. nodes**	**No. edges**	**Average node outgoing degree**	**Average shortest path**	**Cluster learning rate**
Whole connectome	279	5,588 (3,392 intra, 2,196 inter-cluster)	20	2.5	0.00001
11	57	665	11.6	2.17	0.0000843
12	79	1,107	14	2.09	0.00005
13	14	115	8.2	1.52	0.0005
21	74	1,109	14.9	1.97	0.00005
22	55	396	7.2	3.08	0.0001416
11 + 12 +13	150	2,704	18	2.23	0.0000207
21 + 22	129	1,980	15.3	2.34	0.0000283

*Cluster names have hierarchical branch information: 1 (or 2) represents a former branch in the left digit and later branching is called 1 (or two rightward) in the right digit. First, we include information about the whole connectome before the partition, including the number of inter-cluster connections and intra-cluster ones. Then, we include information about the main 5 clusters. Finally, we include also information of the big clusters formed hierarchically from the five main clusters*.

### 2.2. Model Dynamics

Asynchronous state updates are calculated with the following equation:

(1) $$\begin{array}{r} s_{i}\left(t+1\right)=\theta [\overset{N}{\underset{j}{\sum }}\left(\underset{k}{\sum }w_{ijk}\right)s_{j}\left(t\right)] \end{array}$$

where *s*_*i*_ is the state of neuron *i* and *w*_*ijk*_ in the connection weight between neuron *i* and neuron *j* with index *k* (more than one tie with the same direction could arise between *i* and *j*). In a Hopfield network, a node *i* satisfies a constraint with its interaction with node *j* with index *k* if *s*_*i*_*s*_*j*_*w*_*ijk*_ > 0. System energy represents the constraint satisfaction level in the network:

(2) $$\begin{array}{r} E=-\overset{N}{\underset{ijk}{\sum }}w_{ijk}^{O}\left(t\right)s_{i}\left(t\right)s_{j}\left(t\right) \end{array}$$

where $w_{ijk}^{O}$ is the original weight configuration of *w*_*ijk*_, the Hebbian learning changes during the process are managed in another variable.

The self-optimization algorithm consists on the repeating the following sequence of steps, each repetition is called a reset-convergence cycle:

1. Arbitrary assignment of states for the neurons (reset).
2. Convergence of the network for a certain time period, most frequently resulting in an attractor.
3. Application of Hebbian learning.

### 2.3. Introducing Inhibitory Connections

Morales and Froese (2019) explored two different weight configurations with self-optimization: when all connections are excitatory (positive), and when 30% are inhibitory (negative). In order to make the model more realistic, we introduced the inhibitory connections in the second weight configuration (Capano et al., 2015). This is because inhibitory connections are known to have an impact on network dynamics (Brunel, 2000). We found that the network shows a tendency to self-optimize when all connections are excitatory, but the 30% inhibitory connections restrict coordination and constraint satisfaction. Adding inhibitory connections will always have the effect of increasing the difficulty of constraint satisfaction, but it is also likely that this decrease in performance has to do with the fact that we distributed the inhibitory connections in a random fashion without taking the structural organization of the connectome into account. Therefore, we investigated the extent of self-optimization within each of the connectome's functional clusters with 30% inhibitory connections, and also self-optimization of the whole connectome when those inhibitory connections are concentrated between clusters.

More specifically, we run two sets of experiments: (1) self-optimization is run in each isolated cluster separately, and (2) we test for self-optimization in the whole connectome with inhibitory edges only assigned to inter-cluster connections and we monitor each embedded cluster. Since self-optimization in the network is sensitive to its size, we adjusted the learning rate in each isolated cluster in order to make the comparison fairer (see Table 1 for the learning rates). Python code of this simulation is available on GitHub^1^.

## 3. Results

Each experiment consists on the following setup (averaged from 10 different experiments with a different initial random number seed): the network is set to an initial configuration with only positive values and then we performed 1,000 reset-convergence cycles without Hebbian learning. Then, self-optimization is applied using 1,000 reset-convergence cycles that include Hebbian learning. Finally, another 1,000 reset-convergence cycles are applied without Hebbian learning using the learnt configuration obtained so far in order to show its stability. Note that these structural changes accumulated during learning are not directly reflected in the resulting figures. All the energy results shown in the figures were obtained by testing state configurations against the original connectome topology, because this reveals the extent to which the process was able to satisfy the original network constraints.

**Figure 1:**
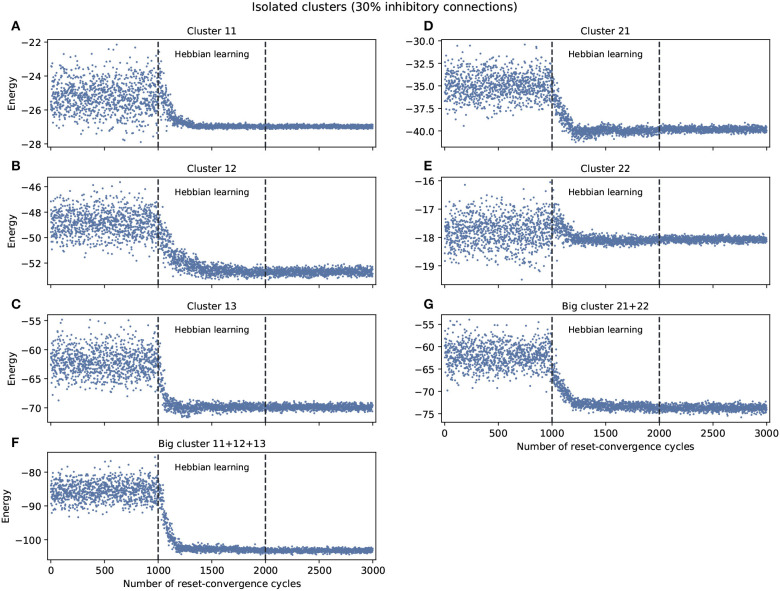
Examples of self-optimization in different *C. elegans* clusters with 30% inhibitory connections; each panel was run separately (independent to the rest of the connectome). The learning rate in each experiment was proportional (regarding the edges) to the one used with the entire connectome and proved to be suitable in the previous work of Morales and Froese (2019). **(A–E)** Correspond to the clusters 11, 12, 13, 21, 22, respectively. **(F,G)** Belong to the two big clusters formed at a higher level from the previous: the chemosensory cluster (11 + 12 + 13) and the mechanosensory one (21 + 22). Each panel was averaged from 10 different experiments and shows the energy of the neuron states in three distinct phases: before learning (1–1,000), during the self-optimization process (1,001–2,000), and after learning (2,001–3,000). Self-optimization can be observed in almost all panels, but tend to remain a diversity of attractors. The difference in y-scale of each panel underline the complexity of the problem to be solved by self-optimization. Energy values averaged in **(A)** before self-optimization produce −25.22 (0.91 SD), during self-optimization −26.71 (0.61 SD), and after self-optimization −26.98 (0.06 SD). In the case of **(E)** we have −17.78 (0.5 SD), −18.01 (0.24 SD), and −18.07 (0.07 SD), respectively.

The experiment shown in Figure 1 explored self-optimization capacity in each isolated cluster, including the big clusters consisting of the join of smaller clusters. Each network tested separately show a tendency to self-coordinate during Hebbian learning, presenting a greater diversity of attractors. Some generalization capacity can also be seen, when a network starts to converge on energy values that were not previously seen during the initial phase. There are two exceptions: cluster 11 converges on a good energy value but one that was already included in the original distribution of energy values, and cluster 22 only converges on an average energy value of the ones previously encountered.

**Figure 2:**
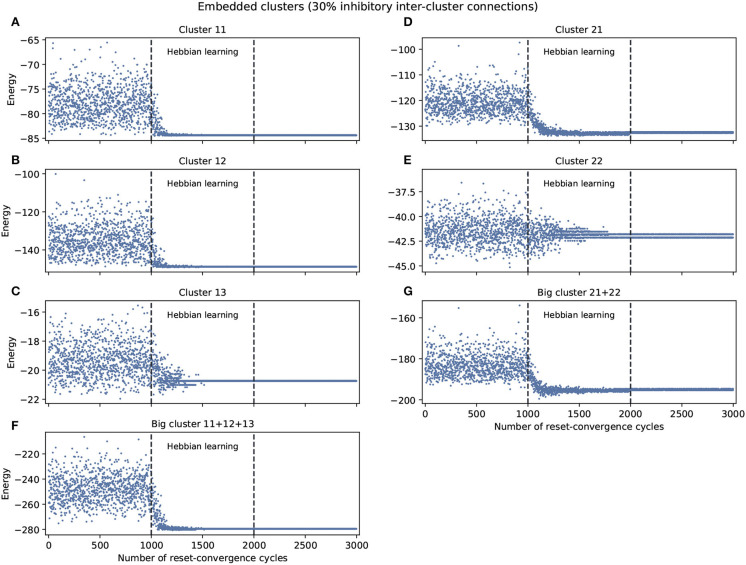
Examples of self-optimization in different *C. elegans* clusters monitored in the context of a single experiment. 30% inhibitory neurons were added arbitrarily only to inter-cluster connections. The learning rate in the experiment was the same to the one used with the entire connectome and proved to be suitable in the previous work of Morales and Froese (2019). **(A-E)** Correspond to the clusters 11, 12, 13, 21, 22, respectively. **(F,G)** Belong to the two big clusters formed at a higher level from the previous: the chemosensory cluster (11+12+13) and the mechanosensory one (21+22). Each panel was averaged from 10 different experiments and shows the energy of the neuron states in three distinct phases: before learning (1-1,000), during the self-optimization process (1,001-2,000), and after learning (2,001-3,000). Self-optimization can be observed in almost all panels, but in this case the global attractors tend to be punctual. Some clusters like 22 represent a complex case for the algorithm. Energy values averaged in **(C)** for cluster 13 before self-optimization produce −19.35(1.13 SD), during self-optimization −20.64(0.42 SD), and after self-optimization −20.74(0 SD).

Figure 2 shows the experiments with 30% inhibitory connections arbitrarily assigned to only inter-cluster connections. We again find a tendency of the energy to decrease and the network to self-optimize, but the capacity for generalization to better previously unseen attractors is less notable. Nevertheless, the embedded clusters converge on better energy values compared to the isolated clusters, although this may be partially because the inhibitory connections were moved to the inter-cluster domain, thereby also decreasing the difficulty of intra-cluster coordination. However, we know that this decrease in intra-cluster complexity is not the whole story because there is one exception: cluster 13 performs worse under these embedded conditions compared to isolated conditions.

**Figure 3:**
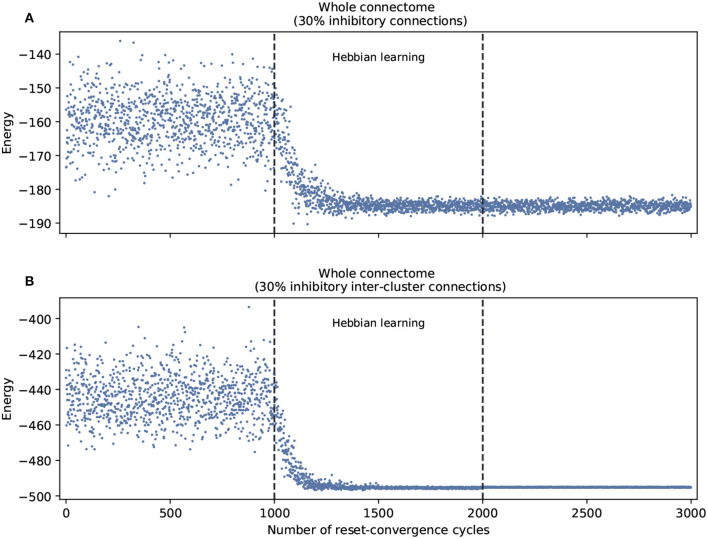
Self-optimization tested in the whole connectome with two different scenarios. **(A)** With 30% inhibitory connections arbitrarily assigned at the beginning of the process. **(B)** With 30% inhibitory connections arbitrarily assigned only in the inter-cluster connections at the beginning of the process. Each panel was averaged from 10 different experiments and shows the energy of the neuron states in three distinct phases: before learning (1–1,000), during the self-optimization process (1,001–2,000), and after learning (2,001–3,000). Both scenarios tend to self-optimize, but the algorithm explores a broader variety of attractors and converges on more optimal patterns of coordination when inhibitory connections are restricted to inter-cluster connections.

This leads us to ask about the performance of self-optimization at the level of the whole connectome. Figure 3 shows that restricting inhibitory connections to the inter-cluster domain has the effect of facilitating the self-optimization process: it now consistently generalizes to a more refined set of energy values that are much lower. This occurs despite the fact that both conditions feature the same overall number of inhibitory connections.

## 4. Discussion

We successfully demonstrated the capacity of self-optimization for the case of the *C. elegans* connectome. Through repeated reset-convergence cycles, the network managed to generalize to previously unseen attractors with better coordination constraint satisfaction. Moreover, we managed to improve on previous work by showing that inhibitory connections do not hinder this process as long as they are concentrated to connections between clusters.

For simplicity, we assigned all inhibitory connections to inter-cluster connections in an arbitrary way. However, in real neural networks it is whole neurons, not isolated connections, that are inhibitory. Future work could therefore further improve the biological realism of our model by taking into account the excitatory or inhibitory functions of the neurotransmitters associated with each of the neurons in the connectome (Riddle et al., 1997; Pereira et al., 2015).

We also note that here we only explored the dynamics of the network in an uncoupled mode. Accordingly, an outstanding challenge is to embed the model of the connectome in whole worm simulations to explore the relationship between coupled and uncoupled dynamics (Izquierdo and Bührmann, 2008; Zarco and Froese, 2018b). So far it is unknown whether self-optimization can also occur when the network is in a coupled mode. Nevertheless, it has been speculated that the uncoupled mode of self-optimization could reflect the prevalent need for sleep among animals (Woodward et al., 2015). If this is on the right track, our model could be developed into a scientific hypothesis to inform current debates about the function of the quiescent state observed in *C. elegans* (Raizen et al., 2008; Trojanowski and Raizen, 2016). Future modeling work could also explore similarities and differences between this proposal and other neural network models of the function of sleep (Hopfield et al., 1983; Fachechi et al., 2019).

One limitation of our work is that the model is not sufficiently realistic compared with living systems and their complex interactions at different levels. We can overcome this limitations by implementing our model under different attractor dynamics like heteroclinic or slow and fast dynamics in synapses (Izhikevich, 2007).

## Data Availability Statement

The raw data supporting the conclusions of this article will be made available by the authors, without undue reservation, to any qualified researcher.

## Author Contributions

All authors listed have made a substantial, direct and intellectual contribution to the work, and approved it for publication.

### Conflict of Interest

The authors declare that the research was conducted in the absence of any commercial or financial relationships that could be construed as a potential conflict of interest.

## Footnotes

^1^https://github.com/aehecatl/self_opt_c_elegans
